# Assessment of TP53 and CDKN2A status as predictive markers of malignant transformation of sinonasal inverted papilloma

**DOI:** 10.1038/s41598-024-64901-z

**Published:** 2024-06-21

**Authors:** Soohyeon Kwon, Jeong-Whun Kim, Eun Sun Kim, Jin Ho Paik, Jin-Haeng Chung, Sung-Woo Cho, Tae-Bin Won, Chae-Seo Rhee, Jee Hye Wee, Hyojin Kim

**Affiliations:** 1 Department of Pathology, Seoul National University Bundang Hospital, Seoul National University College of Medicine, Seongnam, Republic of Korea; 2 Department of Otorhinolaryngology-Head and Neck Surgery, Seoul National University Bundang Hospital, Seoul National University College of Medicine, Seongnam, Republic of Korea; 3 Department of Otorhinolaryngology-Head and Neck Surgery, Hallym University Sacred Heart Hospital, Hallym University College of Medicine, Anyang, Republic of Korea

**Keywords:** Cancer genomics, Head and neck cancer, Tumour biomarkers, Oncogenesis

## Abstract

The mechanism and predictive biomarkers of sinonasal inverted papilloma (IP) transformation into squamous cell carcinoma (SCC) are still unclear. We investigated the genetic mutations involved and the predictive biomarkers. Fourteen patients with SCC arising from IP and six patients with IPs without malignant transformation (sIP) were included. DNA was extracted separately from areas of normal tissue, IP, dysplasia, and SCC. Whole exome sequencing and immunohistochemistry was performed. Major oncogenic mutations were observed in the progression from IP to SCC. The most frequently mutated genes were *TP53* (39%) and *CDKN2A* (27%). Mutations in *TP53* and/or *CDKN2A* were observed in three of six IPs with malignant transformation (cIP); none were observed in sIPs. Tumor mutational burden (TMB) increased from IP to SCC (0.64/Mb, 1.11/Mb, and 1.25 for IP, dysplasia, and SCC, respectively). TMB was higher in the cIPs than in the sIPs (0.64/Mb vs 0.3/Mb). Three cIPs showed a diffuse strong or null pattern in p53, and one showed a total loss of p16, a distinct pattern from sIPs. Our result suggests that *TP53* and *CDKN2A* status can be predictive markers of malignant transformation of IP. Furthermore, immunohistochemistry of p53 and p16 expression can be surrogate markers for *TP53* and *CDKN2A* status.

## Introduction

Sinonasal inverted papillomas (IP) are common benign mucosal neoplasms that occur in the sinonasal tract and are characterized by their inverted growth pattern^1^. Although IPs are classified as benign, they have the potential to progress into squamous cell carcinoma (SCC), with reported rates of malignant transformation ranging from 1.9 to 27%^2^. The development of IP is a complex process that involves various genetic and environmental factors; however, their progression to SCC is poorly understood.

Several studies investigated the molecular mechanisms underlying the tumorigenesis and malignant transformation of IP. Various genetic mutations, including *EGFR, TP53*, *CDKN2A* and *KRAS* mutations, as well as human papillomavirus (HPV) infection have been reported as potential mechanisms of malignant transformation of IP^3–9^. Although one study used whole exome sequencing to find the genetic alterations related to malignant transformation^9^, most other studies used targeted gene panels for next-generation sequencing^3,6–8^, which limited the detection of genetic variants to only the genes included in the panel^10^. Furthermore, targeted sequencing is often performed using only tumor tissue, making it difficult to distinguish between germline and somatic mutations. In addition, there is currently no reliable diagnostic method for predicting malignant transformation. A better understanding of the genetic alterations that contribute to malignant transformation may enable the development of more accurate diagnostic methods and more effective treatment strategies.

In this study, we aimed to investigate the genetic mutations involved in the stepwise progression of IP to SCC and explore potential biomarkers that could predict malignant transformation using whole exome sequencing with matched normal tissue. This approach has the potential to provide a more comprehensive understanding of the genetic alterations that contribute to malignant transformation and to identify new targets for early detection and prevention of IP progression to SCC.

## Materials and methods

### Sample selection and DNA extraction

**Figure 1:**
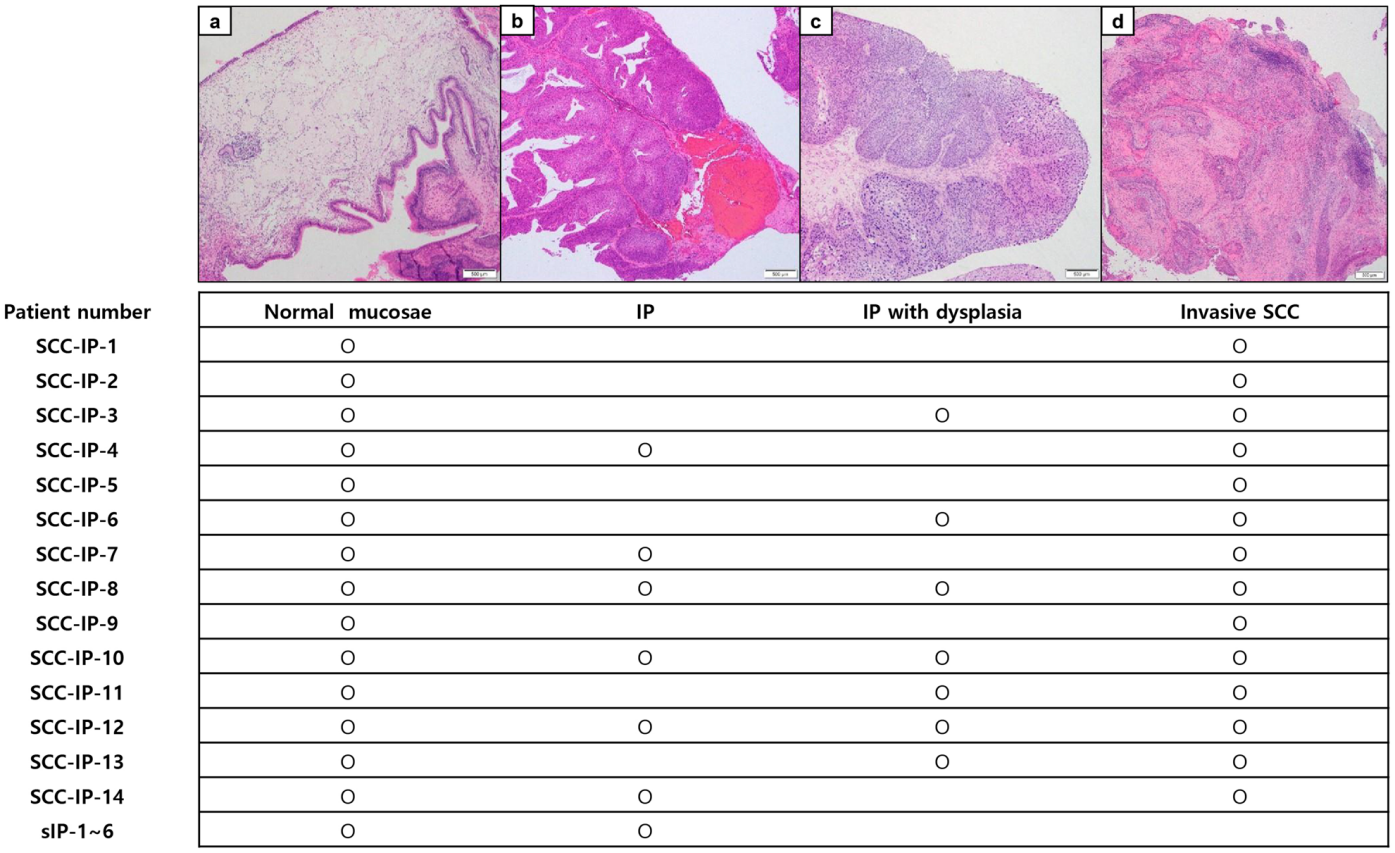
Representative histologic images of normal tissue (**a**), inverted papilloma (**b**), dysplasia (**c**), and squamous cell carcinoma (**d**) with the list of samples included in the sequencing and immunohistochemistry in each patient (× 20 magnification). *IP* inverted papilloma, *SCC* squamous cell carcinoma, *SCC-IP* patients with squamous cell carcinoma arising from inverted papilloma, *sIP* patients with inverted papilloma without malignant transformation.

We included 14 patients who were diagnosed with and treated for SCC arising from IP (SCC-IP) at Seoul National University Bundang Hospital between 2004 and 2020. In addition, six patients who were diagnosed with IP without malignant transformation ("sIP") were included as a comparison group. The hematoxylin and eosin stained slides for each case were reviewed by two pathologists (S.K. and H.K.) to select the areas for sequencing and immunohistochemistry (IHC). In each case, we distinguished each component of normal mucosae, IP, IP with dysplasia, and invasive SCC for macro-dissection. DNA was extracted separately from each component. The list of patients and samples used for sequencing and IHC is shown in Fig. 1. The study protocol was approved by the Institutional Review Board of Seoul National University Bundang Hospital (IRB No. B-2008-630-307), and the study was performed in accordance with the Declaration of Helsinki. Informed consent was obtained from each patient, except for those who died.

### Whole exome sequencing

DNA was extracted using the GeneRead DNA FFPE kit (Qiagen) following the manufacturer's protocol. The quality and quantity of purified DNA were assessed by fluorometry (Qubit, Invitrogen) and gel electrophoresis. Briefly, 200 ng of each sample was ligated to Illumina’s adapters and PCR-amplified. The samples were concentrated to < 1000 ng in 12 μL DW using a SpeedVac machine and hybridized with RNA probes, SureSelectXT Human All Exon V5 at 65 °C 1 min–37 °C 3 s, 60 cycles. After hybridization, the captured targets were pulled down by biotinylated probe/target hybrids using streptavidin-coated magnetic beads (Dynabeads My One Streptavidine T1; Life Technologies Ltd.) and buffers. The selected regions were then PCR-amplified using Illumina PCR primers. Libraries were quantified using the Agilent 4200 Bioanalyzer (Agilent) and KAPA Library Quantification Kit (Kapa Biosystems).

The high quality-libraries were pooled and sequenced on the Illumina NovaSeq6000 platform (Illumina) with 150 bp paired-end by following the manufacturer’s protocols. Image analysis were performed using the NovaSeq6000 control Software version 1.3.1 and the output base calling data was de-multiplexed with bcl2fastq version v2.20.0.422 generating fastQC files. Sequencing reads were aligned to the human reference genome hg19 using Burrows Wheeler Aligner (BWA) (v.0.7.17)^11^. After the alignment of the reads to reference genome, the duplicated reads were further removed using MarkDuplicates in Picard (v.2.20.7). Next, base quality score recalibration (BQSR) process was conducted to adjust the quality score using BaseRecalibrator in Genome Analysis Toolkit (GATK) (v.4.1.3)^12^. For germline and somatic variants calling, GATK^12^ HaplotypeCaller and Mutect2 were utilized, respectively. Further, the LearnReadOrientationModel and FilterMutectCalls of GATK^12^ were employed to filter orientation bias, technical artifacts and sequencing error. In addition to matched normal samples, gnomAD database was utilized to further exclude germline variants. Only variants with a minimum of 10 supporting reads were included. All variants were then annotated using Ensembl VEP v100^13^ considering the effects on transcripts, proteins, and regulatory regions. For known or overlapping variants, allele frequencies and disease or phenotype information were included. For downstream analysis, the variants call format (VCF) files were converted to mutation annotation format (MAF) files using vcf2maf. The variants annotated as PASS were summarized and visualized using R packages maftools^14^.

### p53 and p16 immunohistochemistry

Immunostaining for p53 and p16 were performed using monoclonal mouse anti-human p53 (clone DO-7, 1:1000, Dako, Carpinteria, CA, USA) primary antibody and monoclonal mouse p16 (clone E6H4, CINtec^®^, Ventana Medical Systems, Inc., Tucson, AZ, USA) primary antibody on an automated platform (Benchmark Ultra; Ventana Medical Systems) according to the manufacturer’s instructions. The results were independently interpreted by two pathologists (S.K. and H.K.). P53 expression was classified as diffuse strong positive if there was a diffuse strong nuclear staining in > 80% of tumor cell nuclei, total loss if there was complete absence of staining, and patchy positive if there was variable nuclear staining in 1–80% of tumor cell nuclei^15^. P16 expression was classified as diffuse strong positive if there was a diffuse strong nuclear and cytoplasmic staining in > 90% of tumor cells, total loss if there was complete absence of staining, and patchy positive if there was variable nuclear and/or cytoplasmic staining^16^.

### Human papillomavirus genotyping

HPV status was determined by HPV genotyping. HPV genotyping was performed using peptide nucleic acid probe-based fluorescence melting curve analysis in a real-time PCR system (PANA RealTyper™ HPV Kit, PANAGENE, Daejeon, Republic of Korea) according to the manufacturer’s instructions. It provides a qualitative detection of 40 HPV genotypes, including genotyping information of 20 high-risk types (16, 18, 26, 31, 33, 35, 39, 45, 51, 52, 53, 56, 58, 59, 66, 68, 69, 70, 73, 82) and 2 low-risk types (6, 11), or the presence of 18 low-risk types (30, 32, 34, 40, 42, 43, 44, 54, 55, 61, 62, 67, 74, 81, 83, 84, 87, 90) without genotyping.

## Results

### Clinicopathologic characteristics

The clinicopathologic characteristics of the patients are summarized in Table 1. There was no significant difference in age (63.2 ± 12.1 vs. 62.9 ± 6.9 years), sex, and mean tumor size (4.1 cm vs. 3.4 cm) between the two groups (*p* = 0.935, 0.573, and 0.191, respectively). The five-year survival rate was 71.4% in the SCC-IP group and 100% in the sIP group without statistically significant difference (*p* = 0.763).

**Table 1:** Patient characteristics.

Characteristics	Total (n = 20)	SCC-IP (n = 14)	sIP (n = 6)	*p*-value
Sex				0.573
Male (n, %)	15 (75)	10 (71.4)	5 (83.3)	
Female (n, %)	5 (25)	4 (28.6)	1 (16.7)	
Age (years, mean, SD)	63.1 (10.6)	63.2 (12.1)	62.9 (6.9)	0.935
Tumor size (cm, mean, SD)				
Longest diameter	3.8 (1.2)	4.1 (1.3)	3.4 (0.9)	0.191
Vertical diameter	2.8 (0.8)	2.9 (0.8)	2.7 (0.9)	0.645
Five-year survival (n, %)	16 (80.0)	10 (71.4)	6 (100.0)	0.763

*SCC-IP* squamous cell carcinoma arising from inverted papilloma, *sIP* inverted papilloma without malignant transformation, *n* number, *SD* standard deviation.

### Genomic alteration related with malignant transformation of inverted papilloma

**Figure 2:**
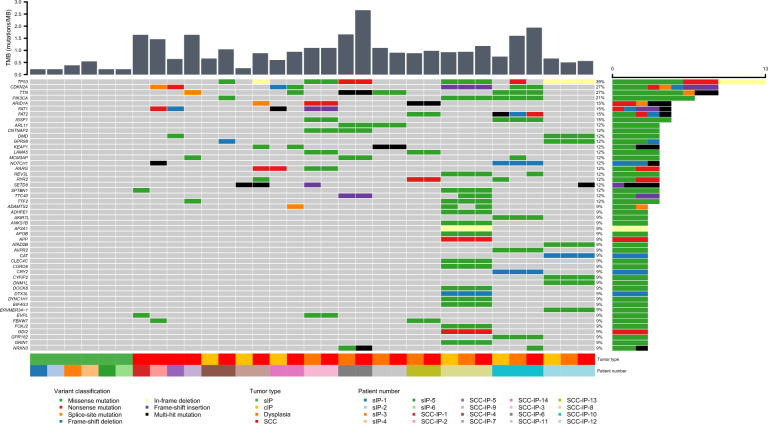
Genetic alterations and tumor mutational burden (TMB) in the benign inverted papilloma and squamous cell carcinoma arising from inverted papilloma (SCC-IP). *sIP* inverted papilloma without malignant transformation, *cIP* inverted papilloma with malignant transformation.

Various single nucleotide variants (SNVs) were identified in SCC-IP group. Top 50 genes that were frequently mutated are shown in Fig. 2. The most common mutated gene was *TP53* (39%), followed by *CDKN2A* (27%), *TTN* (27%), *PIK3CA* (21%), and *ARID1A* (15%). When limited to SCC, the most frequently mutated genes were *TP53* (43%), *CDKN2A* (36%), *TTN* (36%), *ARID1A* (21%), *FAT1* (21%), *KEAP1* (21%), and *PIK3CA* (21%). In contrast, rare mutations were identified in sIP group. The frequencies of commonly mutated genes in each tumor type subgroup are shown in Table 2. The entire list of the mutations can be found in Supplementary Table S1.

**Table 2:** Frequencies of the commonly mutated genes in each tumor type subgroup.

sIP (n = 6)	Freq (%)	cIP (n = 6)	Freq (%)	Dys (n = 7)	Freq (%)	SCC (n = 14)	Freq (%)
		TP53	2 (33%)	TP53	5 (71%)	TP53	6 (43%)
		CDKN2A	2 (33%)	TTN	3 (43%)	CDKN2A	5 (36%)
		PIK3CA	2 (33%)	ARID1A	2 (29%)	TTN	5 (36%)
				ARL11	2 (29%)	ARID1A	3 (21%)
				CDKN2A	2 (29%)	FAT1	3 (21%)
				CNTNAP2	2 (29%)	KEAP1	3 (21%)
				FAT2	2 (29%)	PIK3CA	3 (21%)
				FNBP1L	2 (29%)	A2M	2 (14%)
				IGSF1	2 (29%)	ABTB1	2 (14%)
				LAMA5	2 (29%)	ADAMTS2	2 (14%)
				MCM3AP	2 (29%)	ARL11	2 (14%)
				PIK3CA	2 (29%)	ATG2A	2 (14%)
				SCN7A	2 (29%)	CNTNAP2	2 (14%)
				TTC40	2 (29%)	DMD	2 (14%)
						EVPL	2 (14%)
						FAT2	2 (14%)
						FBXW7	2 (14%)
						FRAS1	2 (14%)
						GPR98	2 (14%)
						HCN1	2 (14%)
						IGSF1	2 (14%)
						KIDINS220	2 (14%)
						KMT2D	2 (14%)
						LAMA5	2 (14%)
						MCM3AP	2 (14%)
						MUC6	2 (14%)
						NDST4	2 (14%)
						NFE2L2	2 (14%)
						NOTCH1	2 (14%)
						NRXN3	2 (14%)
						PCDHA2	2 (14%)
						PDZRN3	2 (14%)
						PHIP	2 (14%)
						PPFIA2	2 (14%)
						RARG	2 (14%)
						REV3L	2 (14%)
						RYR2	2 (14%)
						SETD8	2 (14%)
						SPTBN1	2 (14%)
						TRIM66	2 (14%)
						TTC40	2 (14%)
						TTF2	2 (14%)
						XIRP2	2 (14%)

*sIP* inverted papilloma without malignant transformation, *cIP* inverted papilloma with malignant transformation, *Dys* dysplasia, *SCC* Squamous cell carcinoma, *n* number, *Freq* frequency.

The tumor mutational burden (TMB) was calculated as a number of non-synonymous SNVs and indels per mega base (Mb) (Fig. 2). Mean TMB was higher in IP with malignant transformation (cIP) (0.64/Mb) than in sIP (0.3/Mb), and showed a tendency to gradually increase as cancer progressed within the SCC-IP group (0.64/Mb, 1.11/Mb, and 1.25 for IP, dysplasia, and SCC, respectively) (Fig. 2).

### Multistep analysis of squamous cell carcinoma arising from inverted papilloma focusing on TP53 and CDKN2A

There were six cases which had matched IP and SCC component available for sequencing (SCC-IP-4, 7, 14, 8, 10, and 12). In the case of *TP53* mutations, there were 2/6 (33.3%) cases in which mutations identical to those observed in SCC were already present in the IP, 2/6 (33.3%) cases in which no *TP53* mutation was observed in the IP while SCC had one, and 2/6 (33.3%) cases in which *TP53* mutation was not observed in neither IP nor SCC. For *CDKN2A* mutations, 2/6 (33%) cases showed the same mutations in both IP and SCC, 1/6 (17%) case showed mutations in SCC but not in the IP, and 3/6 (50%) cases showed no mutation in neither IP nor SCC. Taken together, 3/6 (50%) of cIP had the same *TP53* and/or *CDKN2A* mutation as SCC.

In contrast, most of the observed mutations in dysplasia and in SCC were identical. There were seven cases which had matched dysplasia and SCC component available for sequencing (SCC-IP-3, 6, 11, 13, 8, 10, and 12). In all but one case, the mutational status of *TP53* and *CDKN2A* in dysplasia and in SCC was the same. The exceptional case had nonsense *TP53* mutation in dysplasia, but the SCC had no mutation (SCC-IP-10).

### p53 and p16 immunohistochemistry and their correlation with mutational status

**Figure 3:**
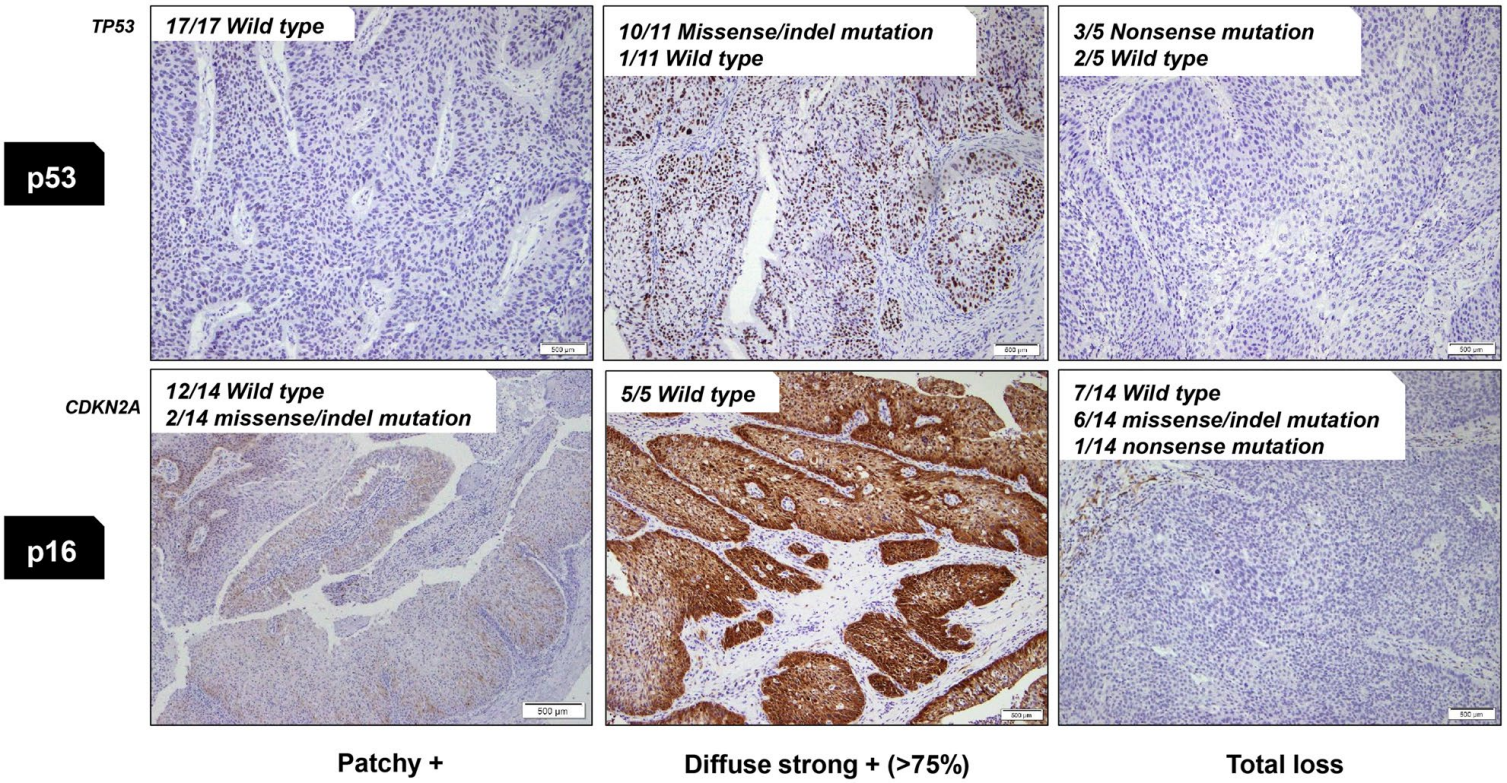
Correlation between *TP53* and *CDKN2A* genetic status and p53 and p16 protein expression.

We first correlated the sequencing results with the IHC results in all samples to confirm the relationship between the presence or absence of *TP53* and *CDKN2A* gene mutations and p53 and p16 protein expression (Table 3 and Fig. 3). Of the 17 samples which showed patchy positivity of p53 protein in IHC, all samples had wild-type *TP53*. When p53 expression was diffuse strong positive in IHC, 10/11 (91%) had missense/indel mutation of *TP53* and 1/11 (9%) had wild type *TP53*. In the samples in which p53 expression showed total loss, nonsense mutation of *TP53* was observed in 3/5 (60%) and wild type in 2/5 (40%).

**Table 3:** p53 and p16 Immunohistochemistry and their correlation with *TP53*, *CDKN2A* mutational status.

Patient number	p53 & *TP53*						p16 & *CDKN2A*						HPV
	IP		Dysplasia		SCC		IP		Dysplasia		SCC		
	p53	*TP53*	p53	*TP53*	p53	*TP53*	p16	*CDKN2A*	p16	*CDKN2A*	p16	*CDKN2A*	
SCC-IP-1					Patchy	Wild type					Patchy	Wild type	
SCC-IP-2					Patchy	Wild type					Total loss	Splice site	
SCC-IP-5					Diffuse strong	Wild type					Total loss	Nonsense	
SCC-IP-9					Patchy	Wild type					Total loss	Wild type	
SCC-IP-4	Patchy	Wild type			Diffuse strong	Missense	Patchy	Wild type			Total loss	Wild type	
SCC-IP-7	Patchy	Wild type			Diffuse strong	In-frame deletion	Patchy	Wild type			Total loss	Wild type	
SCC-IP-14	Patchy	Wild type			Patchy	Wild type	Total loss	Frameshift deletion			Total loss	Missense	
SCC-IP-3			Diffuse strong	Missense	Diffuse strong	Missense			Total loss	Wild type	Total loss	Wild type	
SCC-IP-6			Total loss	Nonsense	Total loss	Nonsense			Patchy	Wild type	Patchy	Wild type	
SCC-IP-11			Patchy	Wild type	Patchy	Wild type			Diffuse strong	Wild type	Diffuse strong	Wild type	Type 16
SCC-IP-13			Patchy	Wild type	Patchy	Wild type			Total loss	Wild type	Total loss	Wild type	
SCC-IP-8	Diffuse strong	Missense	Diffuse strong	Missense	Diffuse strong	Missense	Total loss	Frameshift insertion	Total loss	Frameshift insertion	Total loss	Frameshift insertion	
SCC-IP-10	Total loss	Wild type	Total loss	Nonsense	Total loss	Wild type	Patchy	Wild type	Patchy	Missense	Patchy	Missense	
SCC-IP-12	Diffuse strong	In-frame deletion	Diffuse strong	In-frame deletion	Diffuse strong	In-frame deletion	Diffuse strong	(RB1 frameshift insertion)	Diffuse strong	(RB1 frameshift insertion)	Diffuse strong	(RB1 frameshift insertion)	
sIP-1	Patchy	Wild type					Patchy	Wild type					
sIP-2	Patchy	Wild type					Patchy	Wild type					
sIP-3	Patchy	Wild type					Patchy	Wild type					
sIP-4	Patchy	Wild type					Patchy	Wild type					
sIP-5	Patchy	Wild type					Patchy	Wild type					
sIP-6	Patchy	Wild type					Patchy	Wild type					

*IP* Inverted papilloma, *SCC* squamous cell carcinoma, *sIP* inverted papilloma without malignant transformation, *HPV* human papillomavirus.

For *CDKN2A* and p16 expression, 12/14 (86%) samples had wild type forms and 2/14 (14%) samples had missense/indel mutation of *CDKN2A* in p16 patchy positive tumors. Among five samples which showed diffuse strong positive expression in p16 IHC, all had wild type form of *CDKN2A*, while three had *RB1* frameshift insertion mutation and the other two had high-risk HPV (type 16) infection. The three samples with the *RB1* mutation belong to one case (SCC-IP-12), and the two samples with HPV infection belong to another case (SCC-IP-11). When there was total loss p16 expression, 6/14 (43%) samples had missense/indel mutation, 1/14 (7%) samples had nonsense mutation, and 7/14 (50%) samples had wild type form of *CDKN2A*.

**Figure 4:**
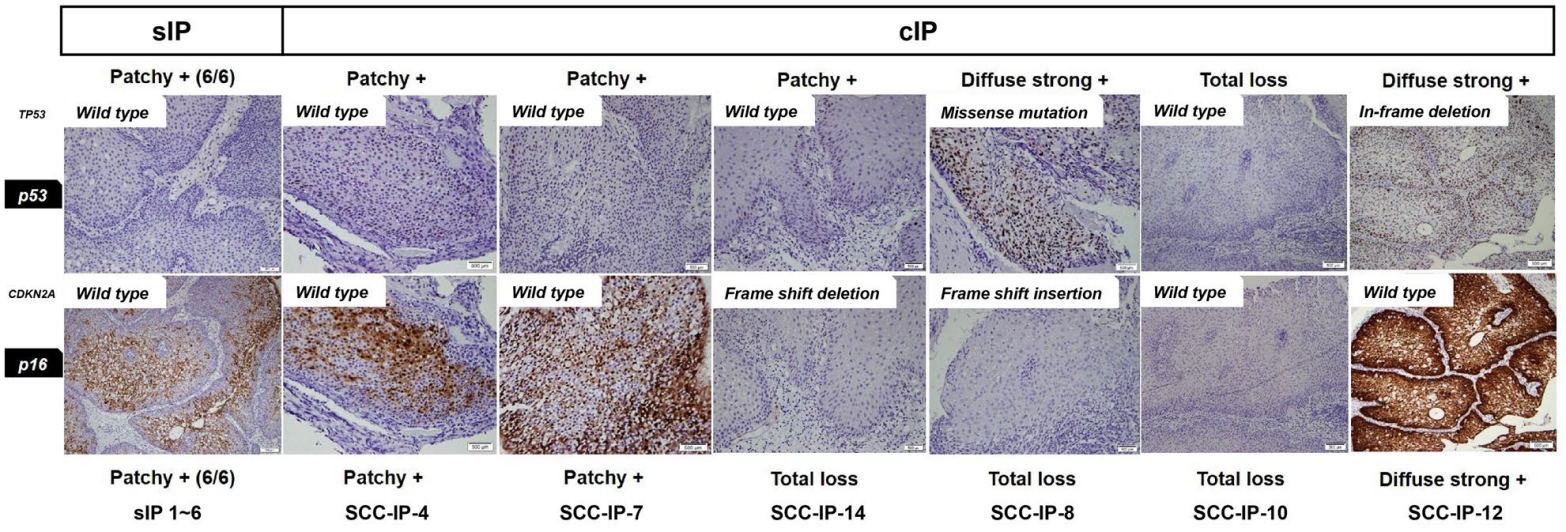
Result of p53, p16 immunohistochemistry, and *TP53* and *CDKN2A* mutations in inverted papilloma with and without malignant transformation (× 20 magnification). *sIP* inverted papilloma without malignant transformation, *cIP* inverted papilloma with malignant transformation.

Focusing on the six cases which had paired IP and SCC component available for sequencing, 4/6 (67%) cases showed aberrant expression (diffuse strong positive or total loss) of p53 and/or p16 in both IP and SCC. The other 2/6 (33%) cases, which showed patchy positive p53 and p16 expression in the IP, exhibited diffuse strong positive expression of p53 in the SCC, which acquired *TP53* mutation during malignant transformation. In contrast, all sIP showed patchy positive p53 and p16 staining. The results of IHC and the mutational status of sIP and cIP are shown in Fig. 4.

### Human papillomavirus infection in squamous cell carcinoma arising from inverted papilloma

High-risk HPV (type 16) was detected in two samples that belonged to one case (SCC-IP-11). Both dysplasia and SCC had HPV infection. As mentioned above, these samples showed diffuse strong positive p16 expression.

## Discussion

In this study, we found that *TP53* and *CDKN2A* could be involved in the early stage of the stepwise progression of IP to SCC and that the assessment of *TP53* and *CDKN2A* status could be a predictive marker of malignant transformation of IP. Moreover, using IHC, we found that p53 and p16 expression could be used as surrogate marker for *TP53* and *CDKN2A* mutational status, respectively, and aberrant expression of p53 and/or p16 could be a predictive marker of malignant transformation of IP.

Both *TP53* and *CDKN2A* are tumor suppressor genes and are observed with high frequency in many tumors^17,18^. Recent studies have shown some conflicting results of *TP53* mutation in cIP. Brown et al. reported that *TP53* mutations and *CDKN2A* mutations/deletions were related to malignant transformation, based on the result that they were observed only in the carcinoma but not in the matched IP^8^. In contrast, Yasukawa et al. reported that most of the *TP53* mutations observed in dysplasia and SCC were already present in IP and there was little difference in mutations observed between IP and SCC^6^. In this study, *TP53* and *CDKN2A* mutations, which were identical to those present in SCC, were observed in 50% (3/6) of cIP, and dysplasia and SCC showed nearly identical mutations. Furthermore, *TP53* and *CDKN2A* mutations were not observed in sIP. This suggests that *TP53* and *CDKN2A* mutations are involved in the early stage of malignant transformation and can be used as biomarkers of early detection of cIP.

There was a strong correlation between *TP53* mutation and the aberrant expression of p53. It was concordant with previous studies on gastric^19^ and ovarian cancers^15^. However, p16 expression did not show a correlation as strong as p53 expression did. When p16 was patchy positive, it was likely that *CDKN2A* was wild type. However, when p16 showed diffuse strong positive staining, there was no *CDKN2A* mutation, while when there was total loss of p16 expression, half of the cases had *CDKN2A* mutation. Traditionally, p16 IHC was used to differentiate high-grade squamous intraepithelial lesion, an HPV-associated squamous lesion of the lower anogenital tract^20^, and as a surrogate marker for HPV testing in HPV-mediated oropharyngeal squamous cell carcinoma^21^. In this context, it was meaningful if p16 expression was manifested as diffuse strong positive, and total loss of p16 would not play a role in the data interpretation. However, recently there has been some reports that the total loss of p16 expression is related to *CDKN2A* mutation, and it is argued that not only the diffuse strong positive expression but also the total loss of p16 expression should be regarded as an abnormal phenotype^16,22^. Moreover, it has been reported that total loss of p16 expression is more frequently seen in SCC-IP than in sIP and is a risk factor for the recurrence of sIP, although the mutational status of *CDKN2A* was not evaluated^23,24^. In this study, 7/9 (78%) samples with *CDKN2A* SNV showed total loss of p16. Conversely, when there was a total loss of p16, *CDKN2A* mutation was found in 7/14 (50%). Additionally, 3/6 (50%) of cIP showed total loss of p16 expression whereas none of the sIP did. Therefore, it is reasonable to consider the total loss of p16 as an aberrant expression and a predictive marker of malignant transformation. In addition, five samples that showed diffuse strong p16 expression had either high-risk HPV infection or *RB1* mutation, which may explain the aberrant p16 expression without *CDKN2A* mutation.

A recent meta-analysis demonstrated that high-risk HPV subtypes 16 and 18 infection was associated with increased risk of malignant transformation of IP^25^. However, the prevalence of high-risk HPV in SCC-IP seems to be low, ranging from 0 to 25%^4,26–29^. In this study, 1 out of 14 SCC-IP patients had high-risk HPV (type 16) and showed diffuse strong positive p16 expression, which implicated the role of high-risk HPV in the pathogenesis of SCC-IP. However, most other SCC-IP specimens did not express high-risk HPV infection. This result was similar to those shown in previous studies in Korea, which did not find HPV infection in any cIP specimen^30,31^. Further studies are needed to clarify the association between HPV infection and malignant transformation of IP.

TMB is defined as the number of mutations per megabase^32^, and whole exome sequencing is generally regarded as the gold standard for TMB measurement^33^. The threshold for high tumor mutational burden (TMB-H) was 10/Mb in KEYNOTE-158 study, based on which the FDA has approved a PD-1 inhibitor, pembrolizumab, for all solid tumors with TMB greater than 10/Mb. Although it is still controversial whether the cut-off value of 10/Mb can be applied universally across all solid tumors^34^, 1.25/Mb, the mean value of TMB of SCC in this study, is much lower than 10/Mb, the cut-off value. Even the highest TMB in this study was 2.66/Mb, which is still considerably low. Therefore, SCC-IP can be regarded as tumors with low TMB. As low-TMB tumors are not suitable candidates for immunotherapy, it is important to identify cIP before it transforms into SCC.

Previous studies have reported frequent *EGFR* mutations in IP, especially exon 20 insertions^3–5,26,29,35^. However, in this study, *EGFR* mutation was not found in any of the cases. This discrepancy can be explained from two points of view: the association with SCC, and geographical distribution. Sahnane et al. reported that *EGFR* mutation was less frequent in SCC-IP (30%) than in sIP (72), and *EGFR*-wild-type IP had higher tendency of malignant transformation than *EGFR*-mutated IP at 5-year follow-up^26^. In this study, 27/33 (82%) samples are from SCC-IP, which might partially explain why all the samples were *EGFR* wild-type. In the aspect of geographical distribution, Yasukawa et al. reported the frequency of *EGFR* mutations to be 20%, 38%, and 0% in IP, dysplasia, and SCC-IP, respectively, in the samples from Hokkaido University Hospital, Japan^6^, whereas Udager et. al reported the frequency of *EGFR* mutations to be 88% in the samples from University of Michigan, USA^3^. As the *EGFR* mutation frequency differs significantly between Japan and USA, it can be assumed that there are difference in geographical distribution. However, Wang et al. reported a high frequency of EGFR mutations (78%) in Chinese patients, although the study included sIP only^35^. Furthermore, Sasaki et al. reported that 90% of sIP and 88% of SCC-IP in Japanese patients harbored *EGFR* mutations^5^, while Cabal et al. found *EGFR* exon 20 mutations in 38% of sIP and 50% of SCC-IP in Spanish patients^29^. Therefore, the difference of the frequency of *EGFR* mutations cannot be explained by geographical distribution alone and further studies are needed.

Nevertheless, this study has a few limitations. This was a retrospective study that included patients from a single institute; therefore, the number of patients was relatively small and we were unable to obtain peripheral blood lymphocytes. The normal mucosae that were used for sequencing were adjacent to IP or SCC and, therefore, may have already harbored some of the mutations of IP or SCC, potentially leading to false negative results. Moreover, the samples for separate sequencing of each component in SCC-IP were obtained synchronously, which may not directly reflect the time course of malignant transformation. However, we sought to compare the differences in genetic mutations between the regions of IP, dysplasia, and SCC tissue in the same patient. In malignant transformation, we suggest that there may be genetic evidence of the same spectrum. In addition, the synchronousness of each component may have some advantages in preoperative biopsy because performing p53 and p16 staining on the preoperative biopsy specimen can help determine the presence of coexisting SCC component and can be clinically helpful during surgical resection in deciding the extent of resection and the necessity of intraoperative frozen examination, etc.

As mentioned above, there was no clinicopathologic difference between sIP and cIP; therefore, additional tests to differentiate between the two are of high importance.

In conclusion, aberrant expression of p53 and/or p16 is indicative of genetic alterations of *TP53* and *CDKN2A*, which could be used as a predictive marker of malignant transformation of IP to SCC.

### Supplementary Information

Supplementary Table S1.

## Data Availability

All analysed and derivative raw data are available from the corresponding author (J.H.W. and H.K.) upon reasonable request.

## References

[1] Lisan Q, Laccourreye O, Bonfils P (2016). Sinonasal inverted papilloma: From diagnosis to treatment. Eur. Ann. Otorhinolaryngol. Head Neck Dis..

[2] Nudell J, Chiosea S, Thompson LD (2014). Carcinoma ex-*Schneiderian papilloma* (malignant transformation): A clinicopathologic and immunophenotypic study of 20 cases combined with a comprehensive review of the literature. Head Neck Pathol..

[3] Udager AM (2015). High-frequency targetable EGFR mutations in sinonasal squamous cell carcinomas arising from inverted sinonasal papilloma. Cancer Res..

[4] Udager AM (2018). Human papillomavirus (HPV) and somatic EGFR mutations are essential, mutually exclusive oncogenic mechanisms for inverted sinonasal papillomas and associated sinonasal squamous cell carcinomas. Ann. Oncol..

[5] Sasaki E, Nishikawa D, Hanai N, Hasegawa Y, Yatabe Y (2018). Sinonasal squamous cell carcinoma and EGFR mutations: A molecular footprint of a benign lesion. Histopathology.

[6] Yasukawa S (2018). Genetic mutation analysis of the malignant transformation of sinonasal inverted papilloma by targeted amplicon sequencing. Int. J. Clin. Oncol..

[7] Uchi R (2021). Genomic sequencing of cancer-related genes in sinonasal squamous cell carcinoma and coexisting inverted papilloma. Anticancer Res..

[8] Brown NA (2021). TP53 mutations and CDKN2A mutations/deletions are highly recurrent molecular alterations in the malignant progression of sinonasal papillomas. Mod. Pathol..

[9] Viitasalo S (2022). Exome sequencing reveals candidate mutations implicated in sinonasal carcinoma and malignant transformation of sinonasal inverted papilloma. Oral. Oncol..

[10] Sun Y (2015). Next-generation diagnostics: Gene panel, exome, or whole genome?. Hum. Mutat..

[11] Li H, Durbin R (2009). Fast and accurate short read alignment with Burrows–Wheeler transform. Bioinformatics.

[12] McKenna A (2010). The genome analysis toolkit: A MapReduce framework for analyzing next-generation DNA sequencing data. Genome Res..

[13] McLaren W (2016). The Ensembl variant effect predictor. Genome Biol..

[14] Mayakonda A, Lin DC, Assenov Y, Plass C, Koeffler HP (2018). Maftools: Efficient and comprehensive analysis of somatic variants in cancer. Genome Res..

[15] Kobel M (2016). Optimized p53 immunohistochemistry is an accurate predictor of TP53 mutation in ovarian carcinoma. J. Pathol. Clin. Res..

[16] Matson DR (2021). A "Null" pattern of p16 immunostaining in endometrial serous carcinoma: An under-recognized and important aberrant staining pattern. Int. J. Gynecol. Pathol..

[17] Olivier M, Hollstein M, Hainaut P (2010). TP53 mutations in human cancers: Origins, consequences, and clinical use. Cold Spring Harb. Perspect. Biol..

[18] Serra, S. & Chetty, R. p16. *J. Clin. Pathol.* **71**, 853–858 (2018). 10.1136/jclinpath-2018-205216 30076191

[19] Hwang HJ (2020). Prediction of TP53 mutations by p53 immunohistochemistry and their prognostic significance in gastric cancer. J. Pathol. Transl. Med..

[20] Darragh TM (2012). The lower anogenital squamous terminology standardization project for HPV-associated lesions: Background and consensus recommendations from the College of American Pathologists and the American Society for Colposcopy and Cervical Pathology. Arch. Pathol. Lab. Med..

[21] El-Naggar AK, Westra WH (2012). p16 expression as a surrogate marker for HPV-related oropharyngeal carcinoma: A guide for interpretative relevance and consistency. Head Neck.

[22] Schaefer IM (2017). Abnormal p53 and p16 staining patterns distinguish uterine leiomyosarcoma from inflammatory myofibroblastic tumour. Histopathology.

[23] Lin GC (2013). Epidermal growth factor receptor, p16, cyclin D1, and p53 staining patterns for inverted papilloma. Int. Forum Allergy Rhinol..

[24] Menendez M (2022). Loss of p16 expression is a risk factor for recurrence in sinonasal inverted papilloma. Rhinology.

[25] McCormick JP, Suh JD, Lee JT, Wells C, Wang MB (2022). Role of high-risk HPV detected by PCR in malignant sinonasal inverted papilloma: A meta-analysis. Laryngoscope.

[26] Sahnane N (2019). Comprehensive analysis of HPV infection, EGFR exon 20 mutations and LINE1 hypomethylation as risk factors for malignant transformation of sinonasal-inverted papilloma to squamous cell carcinoma. Int. J. Cancer.

[27] Rooper LM, Bishop JA, Westra WH (2017). Transcriptionally active high-risk human papillomavirus is not a common etiologic agent in the malignant transformation of inverted schneiderian papillomas. Head Neck Pathol..

[28] Mehrad M (2020). Transcriptionally active HPV and targetable EGFR mutations in sinonasal inverted papilloma: An association between low-risk HPV, condylomatous morphology, and cancer risk?. Am. J. Surg. Pathol..

[29] Cabal VN (2020). EGFR mutation and HPV infection in sinonasal inverted papilloma and squamous cell carcinoma. Rhinology.

[30] Kim JY, Yoon JK, Citardi MJ, Batra PS, Roh HJ (2007). The prevalence of human papilloma virus infection in sinonasal inverted papilloma specimens classified by histological grade. Am. J. Rhinol..

[31] Hwang CS, Yang HS, Hong MK (1998). Detection of human papillomavirus (HPV) in sinonasal inverted papillomas using polymerase chain reaction (PCR). Am. J. Rhinol..

[32] Merino DM (2020). Establishing guidelines to harmonize tumor mutational burden (TMB): In silico assessment of variation in TMB quantification across diagnostic platforms: Phase I of the Friends of Cancer Research TMB Harmonization Project. J. Immunother. Cancer..

[33] Doig KD, Fellowes A, Scott P, Fox SB (2022). Tumour mutational burden: An overview for pathologists. Pathology.

[34] Strickler JH, Hanks BA, Khasraw M (2021). Tumor mutational burden as a predictor of immunotherapy response: Is more always better?. Clin. Cancer Res..

[35] Wang H (2019). EGFR and KRAS mutations in Chinese patients with sinonasal inverted papilloma and oncocytic papilloma. Histopathology.

