# Author Correction: Assessment of TP53 and CDKN2A status as predictive markers of malignant transformation of sinonasal inverted papilloma

**DOI:** 10.1038/s41598-024-67395-x

**Published:** 2024-07-17

**Authors:** Soohyeon Kwon, Jeong-Whun Kim, Eun Sun Kim, Jin Ho Paik, Jin-Haeng Chung, Sung-Woo Cho, Tae-Bin Won, Chae-Seo Rhee, Jee Hye Wee, Hyojin Kim

**Affiliations:** 1 Department of Pathology, Seoul National University Bundang Hospital, Seoul National University College of Medicine, Seongnam, Republic of Korea; 2 Department of Otorhinolaryngology-Head and Neck Surgery, Seoul National University Bundang Hospital, Seoul National University College of Medicine, Seongnam, Republic of Korea; 3 Department of Otorhinolaryngology-Head and Neck Surgery, Hallym University Sacred Heart Hospital, Hallym University College of Medicine, Anyang, Republic of Korea

Correction to: *Scientific Reports* 10.1038/s41598-024-64901-z, published online 21 June 2024

The original version of the Article contained an error in the Author Information section.

“These authors contributed equally: Soohyeon Kwon, Jeong-Whun Kim, Jee Hye Wee and Hyojin Kim.”

now reads:

“These authors contributed equally: Soohyeon Kwon and Jeong-Whun Kim.

These authors jointly supervised this work: Jee Hye Wee and Hyojin Kim.”

The original Article has been corrected.

